# Novel implications of a strictly monomorphic (GCC) repeat in the human PRKACB gene

**DOI:** 10.1038/s41598-021-99932-3

**Published:** 2021-10-19

**Authors:** Safoura Khamse, Zahra Jafarian, Ali Bozorgmehr, Mostafa Tavakoli, Hossein Afshar, Maryam Keshavarz, Razieh Moayedi, Mina Ohadi

**Affiliations:** 1 Iranian Research Center on Aging, University of Social Welfare and Rehabilitation Sciences, Tehran, Iran; 2 Research Center for Addiction and Risky Behaviors, Iran University of Medical Sciences, Tehran, Iran; 3 Department of Applied Mathematics, Faculty of Mathematical Sciences, Ferdowsi University of Mashhad, P.O. Box 1159, Mashhad, 91775 Iran; 4 Translational Biogerontology, German Center for Neurodegenerative Diseases (Deutsches Zentrum Fur Neurodegenerative Erkrankungen, (DZNE)), Sigmund-Freud-str. 27, Bonn, Germany; 5 Department of Economics, Vrije Universiteit Amsterdam, Amsterdam, The Netherlands

**Keywords:** DNA, Structural biology, Evolution, Genetics, Molecular biology, Neuroscience

## Abstract

*PRKACB* (Protein Kinase CAMP-Activated Catalytic Subunit Beta) is predominantly expressed in the brain, and regulation of this gene links to neuroprotective effects against tau and Aβ-induced toxicity. Here we studied a (GCC)-repeat spanning the core promoter and 5′ UTR of this gene in 300 human subjects, consisting of late-onset neurocognitive disorder (NCD) (N = 150) and controls (N = 150). We also implemented several models to study the impact of this repeat on the three-dimensional (3D) structure of DNA. While the *PRKACB* (GCC)-repeat was strictly monomorphic at 7-repeats, we detected two 7/8 genotypes only in the NCD group. In all examined models, the (GCC)7 and its periodicals had the least range of divergence variation on the 3D structure of DNA in comparison to the 8-repeat periodicals and several hypothetical repeat lengths. A similar inert effect on the 3D structure was not detected in other classes of short tandem repeats (STRs) such as GA and CA repeats. In conclusion, we report monomorphism of a long (GCC)-repeat in the *PRKACB* gene in human, its inert effect on DNA structure, and enriched divergence in late-onset NCD. This is the first indication of natural selection for a monomorphic (GCC)-repeat, which probably evolved to function as an “epigenetic knob”, without changing the regional DNA structure.

## Introduction

Short tandem repeats (STRs) are the most polymorphic genetic elements in the vertebrate genomes. Because of their polymorphic nature and plasticity, these elements provide an efficient source of variation at the inter- and intraspecies levels^1–6^. Accumulating evidence indicates that certain STRs may be associated with selective advantage in human and other species^7–10^. Among the long STRs spanning the core promoter and 5′ untranslated region (UTR) of human protein-coding genes, the protein kinase cAMP-activated catalytic subunit beta (*PRKACB*) gene contains a (GCC)-repeat of 7-repeats^11^. Across human tissues, *PRKACB* has the highest level of expression in the brain^12^ (https://www.proteinatlas.org/ENSG00000142875-PRKACB/tissue). Furthermore, in comparison with fourteen non-human primates, the brain expression of this gene reaches highest quantity in human (https://www.ncbi.nlm.nih.gov/ieb/research/acembly/av.cgi?db=human&term=PRKACB&submit=Go). The kinase encoded by *PRKACB* is involved in tau phosphorylation at Alzheimer’s disease (AD)-related sites, and regulation of this gene has been linked to neuroprotective effects against tau and Aβ-induced toxicity^13,14^.

The length of the *PRKACB* (GCC)-repeat in human is among the rare long lengths for this type of STR^7^. We hypothesized that this length may be of selective advantage to human. In view of the pathways in which *PRKACB* is involved in, this (GCC)-repeat may have a role in the higher order brain functions, such as cognition. Here, we studied the (GCC)-repeat in a sample of human subjects, consisting of late-onset neurocognitive disorder (NCD) and controls, and implemented several models to analyze its impact on DNA three-dimensional (3D) structure.

## Materials and methods

### Subjects

Three hundred unrelated Iranian subjects, consisting of late-onset NCD patients (N = 150) and controls (N = 150) were recruited from the provinces of Tehran, Qazvin, and Rasht. In each NCD case, the Persian version of the Abbreviated Mental Test Score (AMTS)^15,16^ was implemented (AMTS of < 7 was an inclusion criterion for NCD), medical records were reviewed in all participants, and CT-scans were taken where possible. The AMTS is currently one of the most accurate primary screening instruments to increase the probability of NCD^17^, and the Persian version of the AMTS is a valid cognitive assessment tool for older Iranian adults, which can be used for NCD screening in Iran^15^. The control group was selected based on cognitive AMTS of > 7, lack of major medical history, and CT-scan where possible. The cases and controls were matched based on age, gender, and residential district. The subjects' informed consent was obtained (from their guardians where necessary) and their identities remained confidential throughout the study. The research was approved by the Ethics Committee of the University of Social Welfare and Rehabilitation Sciences, Tehran, Iran, and was consistent with the principles outlined in an internationally recognized standard for the ethical conduct of human research. All methods were performed in accordance with the relevant guidelines and regulations.

### Allele and genotype analysis of the *PRKACB* (GCC)-repeat

Genomic DNA was obtained from peripheral blood using a standard salting out method. PCR reactions for the amplification of the *PRKACB* (GCC)-repeat were set up with the following primers Forward: CGCCTGCGAAGATACAGTC, Reverse: CAACTCACCGCTCTCCAC.

PCR reactions were carried out using High GC buffer (30% of the final volume per PCR reaction) in a thermocycler (PEQSTAR, model: PEQLAB) under the following conditions: 95 °C for 4 min, 35 cycles of denaturation at 94 °C for 30 s, annealing for 30 s at 58 °C and extension at 72 °C for 40 s, and a final extension of 72 °C for 5 min. All the samples included in this study were sequenced by the forward primer, using an ABI PRISM 3130 DNA sequencer. The divergent genotypes were sequenced by the reverse primer as well (Supplementary 1).

### Analysis of the *PRKACB* (GCC)7 across vertebrates

The interval between + 1 and + 100 of the transcription start site (TSS) of the *PRKACB* was searched across several orders of vertebrates based on the Ensembl Release 102 (https://asia.ensembl.org/index.html). Alignment was performed using CodonCode Aligner 9.0.1.

### In silico DNA reconstruction of various (GCC)-repeat lengths in human

The DNA 3D structure across the human *PRKACB* (GCC)-repeat was studied using several models, including AA-Wedge, DNase, Bolshoy, Cacchione, and Calladine (Supplementary 2)^18–23^. For each reconstruction, the 100 nucleotide flanking sequences to the repeats were also included. As an instance, based on the twist, roll and tilt, the AA-Wedge model predicts experimental A-tract curvature as measured by gel retardation and cyclization kinetics^18^. In comparison to a number of existing DNA predicting models, such as the Crothers, Dickerson, Jernigan, Tung-Harvey, and Zhurkin, the AA-Wedge model has been reported as the most consistent and accurate^19^. Following obtaining the coordinates of the nucleotides in a 3D space, the DNA structure was visualized using plot3D package in R software^24^.

Furthermore, three additional STRs, *SMAD9* (GCC)-STR, *RASGEF1* (GGC)-STR, and *GPM6B* (GA)-STR, were reconstructed according to the AA-Wedge model^18,19^. The additional genes were studied as sample, for comparison between GCC and non-GCC STR effects.

### Divergence calculation across DNA constructs of various periodical (GCC)-repeat lengths

Let $\left( {x_{i} ,y_{i} ,z_{i} } \right)$ for $i = 1, 2, \ldots , n$ be coordinates of points for each repeat, in the first step we scaled these coordinates as follows

$$\begin{aligned} X_{i} & = \frac{{x_{i} - min\left( {x_{i} } \right)}}{{\left( {x_{i} } \right) - min\left( {x_{i} } \right)}} \quad i = 1,2, \ldots ,n \\ Y_{i} & = \frac{{y_{i} - min\left( {y_{i} } \right)}}{{\left( {y_{i} } \right) - min\left( {y_{i} } \right)}} \quad i = 1,2, \ldots ,n \\ Z_{i} & = \frac{{z_{i} - min\left( {z_{i} } \right)}}{{\left( {z_{i} } \right) - min\left( {z_{i} } \right)}}\quad i = 1,2, \ldots ,n \\ \end{aligned}$$

Parametric equations of a segment line that passes through $\left( {X_{i} ,Y_{i} ,Z_{i} } \right)$’s were

$$\{ X\left( t \right) = \mathop \sum \limits_{i = 1}^{n - 1} (X_{i} - \left( {t\left( {n - 1} \right) - \left( {i - 1} \right)} \right)\left( {X_{i + 1} - X_{i} } \right)I_{{\left[ {\frac{i - 1}{{n - 1}},\frac{i}{n - 1}} \right)}} \left( t \right) Y\left( t \right) = \mathop \sum \limits_{i = 1}^{n - 1} (Y_{i} - \left( {t\left( {n - 1} \right) - \left( {i - 1} \right)} \right)\left( {Y_{i + 1} - Y_{i} } \right)I_{{\left[ {\frac{i - 1}{{n - 1}}, \frac{i}{n - 1}} \right)}} \left( t \right) Z\left( t \right) = \mathop \sum \limits_{i = 1}^{n - 1} (Z_{i} - \left( {t\left( {n - 1} \right) - \left( {i - 1} \right)} \right)\left( {Z_{i + 1} - Z_{i} } \right)I_{{\left[ {\frac{i - 1}{{n - 1}},\frac{i}{n - 1}} \right)}} \left( t \right)\quad t \in \left[ {0,1} \right] ,$$

where $I_{{\left[ {a,b} \right)}} \left( t \right) = 1$ if $t \in \left[ {a,b} \right)$ and $I_{{\left[ {a,b} \right)}} \left( t \right) = 0$ if $t \notin \left[ {a,b} \right)$. To measure similarity between two scaled lines, we defined integrated Euclidean distance (IED). Let $\left( {X\left( t \right),Y\left( t \right),Z\left( t \right)} \right)$ and $\left( {X^{\prime } \left( t \right),Y^{\prime } \left( t \right),Z^{\prime } \left( t \right)} \right)$ for $t \in \left[ {0,1} \right]$ are two lines then IED is as follows

$$IED = \mathop \int \limits_{0}^{1} \sqrt {\left( {X\left( t \right) - X^{\prime } \left( t \right)} \right)^{2} + \left( {Y\left( t \right) - Y^{\prime } \left( t \right)} \right)^{2} + \left( {Z\left( t \right) - Z^{\prime } \left( t \right)} \right)^{2} } dt.$$

)i.j)-th elements revealed the divergence scores obtained from the above method between the i-th and j-th diagrams.

The accuracy of data was validated by two-by-two comparison of constructs of identical lengths.

### Gene network reconstruction

The STRING database (https://string-db.org) was used to find the interactions of *PRKACB* with other genes. STRING is a biological database of known molecular interactions containing information from experimental, computationally predicted, and public text collections^25^. The minimum required interaction score was set at 0.7, and the maximum number of interactions was set at 20. To ensure the most important and reliable interactions, we selected only data from experimental studies as the active interaction sources. Subsequently, the interactive network was reconstructed, using Cytoscape version 3.8.2 and according to the interactions found^26^.

## Results

### Predominant monomorphism of the *PRKACB* (GCC)-repeat in human, at 7-repeats, and divergence from monomorphism in two patients afflicted with late-onset NCD

**Figure 1:**
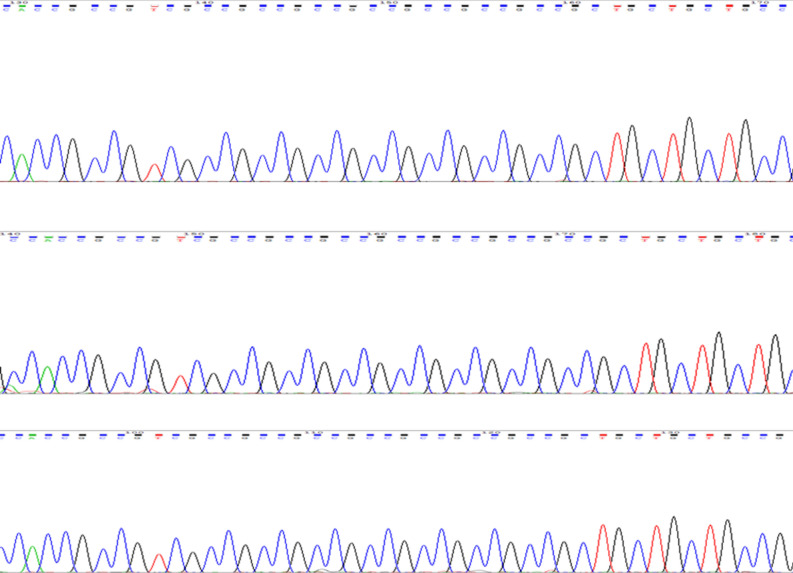
Strict monomorphism of the *PRKACB* (GCC)-repeat, at 7-repeats, in human. Only four samples are represented as examples.

**Figure 2:**
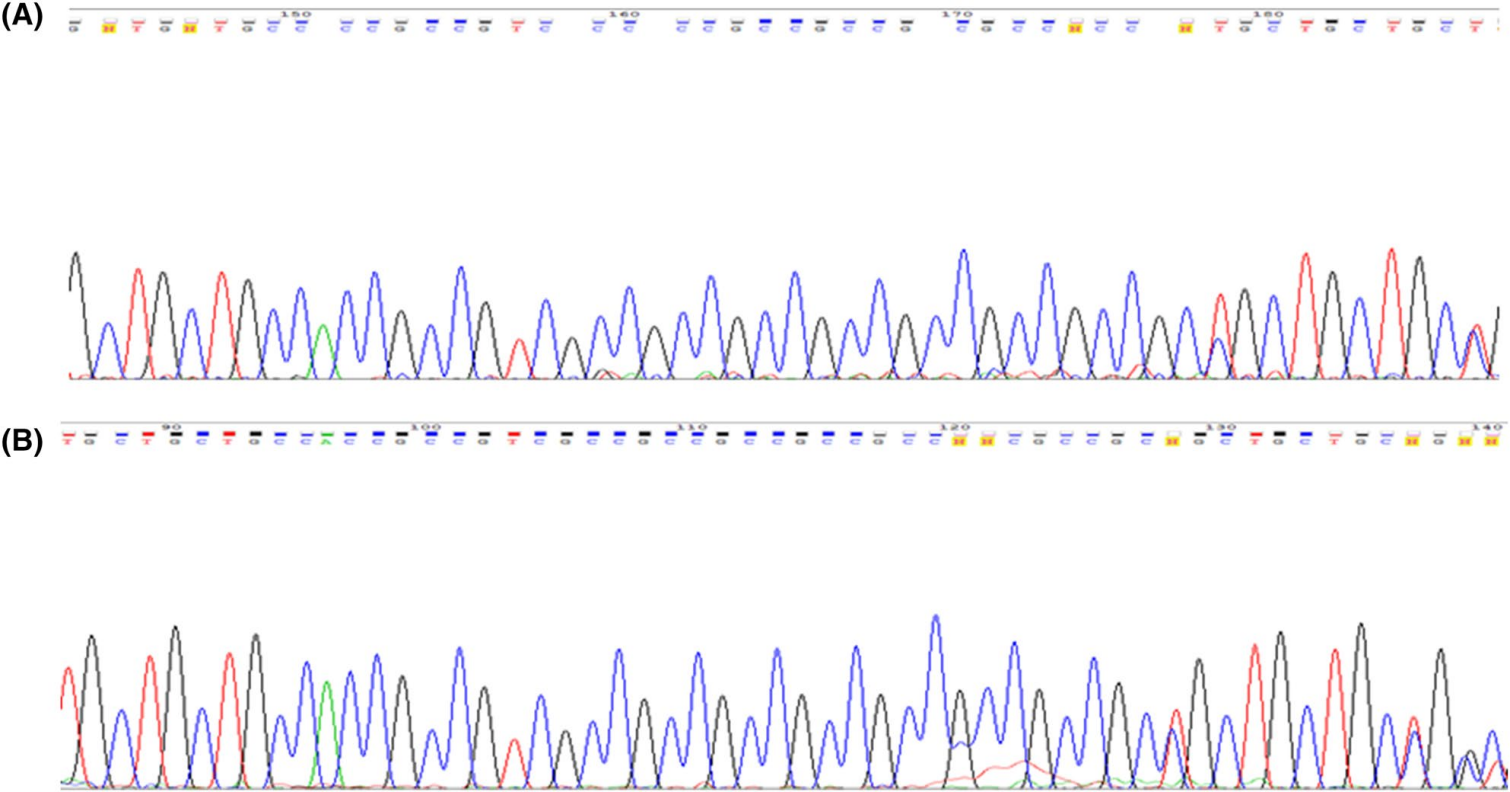
Two cases of late-onset NCD with 7/8 genotypes.

We sequenced the *PRKACB* (GCC)-repeat in 300 human subjects, and found strict monomorphism of this STR at 7-repeats in this human sample (Fig. 1). Exceptions included two 7/8 genotypes in two patients with late-onset NCD (Fig. 2). The two patients harboring the 7/8 genotypes were females of 78 (Patient 1) and 83 (Patient 2) years of age and AMTS of 4 and 3, respectively.

In patient 1 the progression of neurocognitive symptoms was gradual, strengthening the possible diagnosis of AD. In patient 2, symptoms of neurocognitive impairment including aphonia and dementia occurred abruptly and subsequent to a cerebrovascular accident two years before the interview, strengthening the possible diagnosis of vascular dementia. Other causes of late-onset neurological disorders were ruled out in both patients by neurologists.

### Status of the *PRKACB* (GCC)-repeat across vertebrates

**Figure 3:**
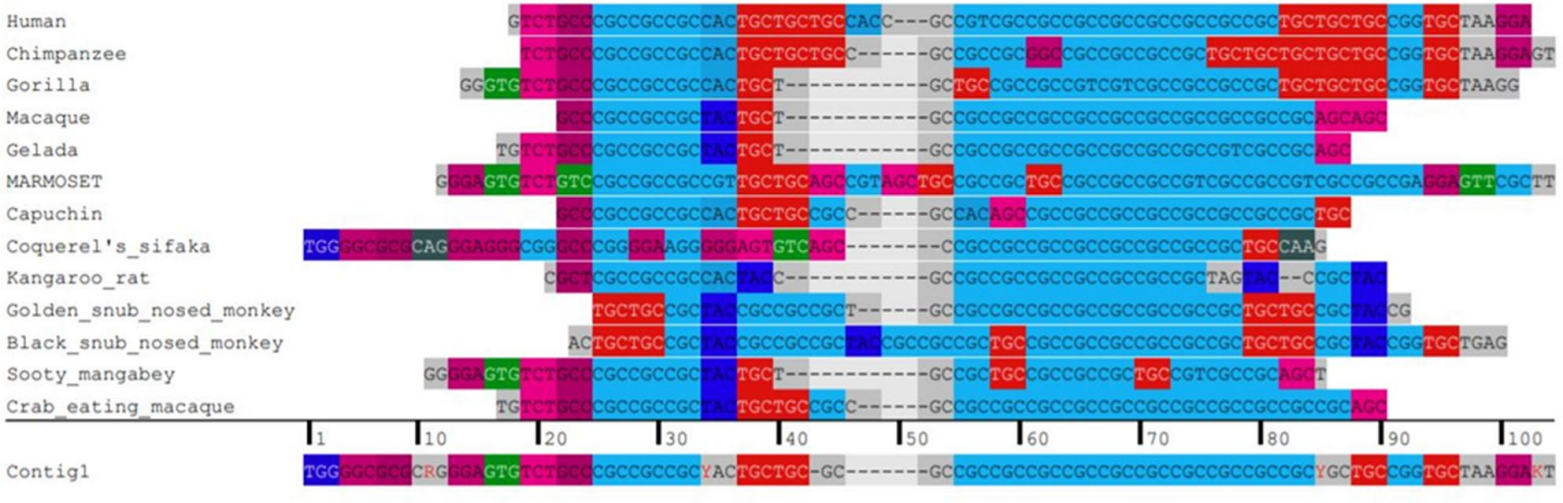
Sequence alignment of the *PRKACB* region encompassing the (GCC)-repeat in various primates. Each species revealed complex and unique stretches of (GCC)-repeats. Contig1 indicates the possible ancestral sequence across the selected species. Triplet nucleotides which may be remnants of GCC sequences are detectable in the ancestral sequence.

The *PRKACB* (GCC)-repeat was highly conserved in numerous orders of vertebrates (Table 1). This repeat probably emerged in Birds and Reptiles, as we did not detect it in Amphibians, Fish, and other eukaryotes. In human, the interval encompassing the + 1 to + 100 to the TSS contains two (GCC)-repeats of (GCC)3 and (GCC)7 formula (Table 1). We detected a long ancestral trace of (GCC)-repeats, identifiable by triplet nucleotides of (GCC) or non-(GCC) in various species (Fig. 3). This long trace of (GCC) resulted in complex and unique (GCC) blocks in each species.

**Table 1:** Status of the *PRKACB* (GCC)-repeat across vertebrates.

Species	Transcript ID	STR formula	Species	Transcript ID	STR formula
Primates			Leopard	ENSPPRT00000024708.1	(GCC)3
Human	ENST00000370689.6	(GCC)3 (GCC)7	Dingo	–	–
Bonobo	–	–	Shrew	–	–
Chimpanzee	ENSPTRT00000109165.1	(GCC)3 (GCC)4			
Gorilla	ENSGGOT00000045976.1	(GCC)3 (GCC)3	Lamprey	–	–
Orangutan	–	–	Coelacanth	–	–
Gibbon	ENSNLET00000012651.2	(GCC)2	Elephant shark	ENSCMIT00000019194.1	(GCC)2
Crab-eating macaque	ENSMFAT00000033375.1	(GCC)2 (GCC)13	Hagfish	–	–
Macaque	ENSMMUT00000076404.2	(GCC)2 (GCC)10	Afrotheria		
Golden snub-nosed monkey	ENSRROT00000025377.1	(GCC)2 (GCC)8	Elephent	–	–
Gelada	ENSTGET00000029662.1	(GCC)2 (GCC)8	Hyrax	–	–
Mouse lemur	ENSMICT00000059697.1	(GCC)5	Lesser hedgehog tenrec	–	–
Vervet-AGM	–	–	Xenatrthra		
Marmoset	ENSCJAT00000115300.1	(GCC)3 (GCC)4	Armadilo	–	–
Sooty mangabey	ENSCATT00000063374.1	(GCC)2 (GCC)3	Sloth	–	–
Drill	ENSMLET00000056274.1	(GCC)7 (GCC)1	Other mammals		
Pig-tailed macaque	ENSMNET00000056095.1	(GCC)2 (GCC)6	Common wombat	–	–
Angola colobus	–	–	Koala	–	–
Ugandan red Colobus	ENSPTET00000046092.1	(GCC)11	Opossum	ENSMODT00000083179.1	(GCC)2
Black snub-nosed monkey	ENSRBIT00000058758.1	(GCC)2 (GCC)6	Platypus	ENSOANT00000057701.1	(GCC)2
Ma's night monkey	ENSANAT00000031038.1	(GCC)3 (GCC)2	Birds and reptiles		
Capuchin	ENSCCAT00000025557.1	(GCC)3 (GCC)8	Bengalese finch	ENSLSDT00000018202.1	(GCC)2 (GCC)3
Bolivian squirrel monkey	ENSSBOT00000050020.1	(GCC)2	Blue tit	–	–
Tarsier	–	–	Burrowing owl	–	–
Greater bamboo lemur	ENSPSMT00000037282.1	(GCC)5	Chicken	ENSGALT00000062352.2	(GCC)3
Coquerel's sifaka	ENSPCOT00000017095.1	(GCC)7	Common canary	–	–
Bushbaby	–	–	Golden eagle	ENSACCT00020005753.1	(GCC)4
Rodent			Kakapo	ENSSHBT00005016149.1	(GCC)4
Mouse	ENSMUST00000102515.9	(GCC)3 (GCC)4	Anole lizard	ENSACAT00000030613.1	(GCC)4
Long-tailed chinchilla	ENSCLAT00000008409.1	(GCC)6	Eastern brown snake	ENSPTXT00000009104.1	(GCC)2
Kangaroo rat	ENSDORT00000033480.1	(GCC)3 (GCC)7	Amphibians		
Naked mole-rat female	ENSHGLT00000016208.1	(GCC)5	Tropical clawed frog	–	–
Rabbit	ENSOCUT00000051257.1	(GCC)5 (GCC)10	Fish		
Laurasiatheria			Asian bonytongue	–	–
Cat	ENSFCAT00000050575.2	(GCC)3	Zebrafish	–	–
Cow	ENSBTAT00000074614.1	(GCC)4 (GCC)5	Common crap	–	–
Goat	–	–	Zig-zag eel	–	–
Blue whale	ENSBMST00010016111.1	(GCC)3	Other eukaryotes		
Horse	ENSECAT00000063662.1	(GCC)8	Saccharomyces cerevisiae	–	–
Siberian musk deer	ENSMMST00000013945.1	(GCC)6	Drosophila melanogaster	–	–
Red Fox	ENSVVUT00000055345.1	(GCC)2	Caenorhabditis elegans	–	–
PIG	–	–	Other cordates		
Dog	–	–	C.intestinalis	–	–
Panda	–	–	C.savignyi	–	–

### In silico reconstruction of the human *PRKACB* 5′ UTR sequence encompassing the (GCC)-repeat

**Figure 4:**
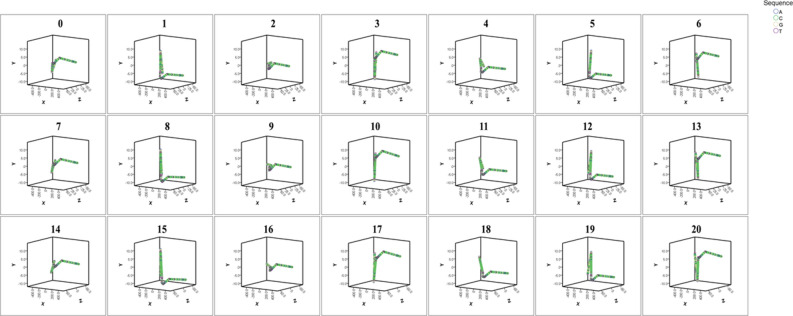
3D construction of human DNA encompassing the *PRKACB* (GCC)-repeat based on the AA-Wedge model. Striking similarity was detected in repeats where subtraction or addition of 7-repeats was included.

We detected only two alleles in the human population studied (7 and 8-repeats). We reconstructed DNA for those repeat lengths and their periodicals in order to analyze their effect on the 3D structure of the region. Across all studied models, the (GCC)7 periodicals e.g. (0, 7, 14), (1, 8, 15), (2, 9, 16), had a significantly less effect on the regional DNA structure in comparison to the 8-repeat periodicals e.g. (0, 8, 16), (1, 9, 17), (2, 10, 18) (Fig. 4, Table 2, Supplementary 2). While the 7-repeat periodicals were strictly consistent in respect of having the least amount of divergence variation, all other repeat length periodicals, including the 8-repeat, were noncontinuous and interrupted by high divergence scores.

**Table 2:**
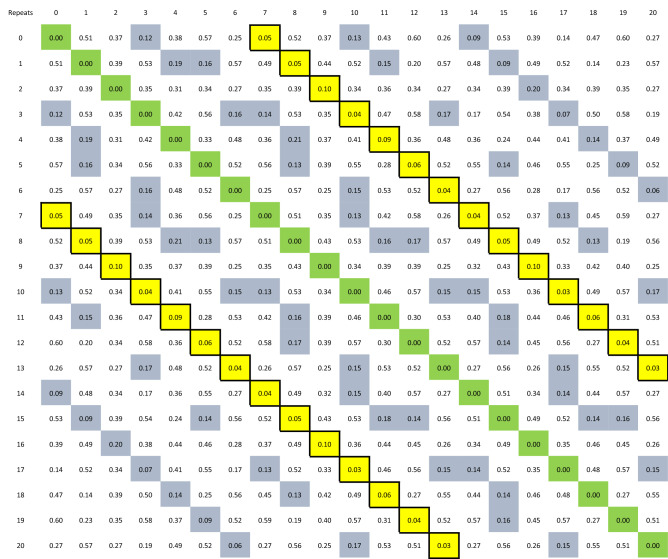
Divergence scores of existing (7 and 8-repeat) and hypothetical *PRKACB* repeat periodicals in human.

The highlighted areas represent the 30 percentile of the entire scores (the lowest 30% of the entire scores). The 7-repeat periodical (solid yellow cells) revealed continuous and strictly narrow divergence score variation in comparison to the 8-repeat and all other periodicals. Only the 7 and 8 repeats were detected in the human population studied.

To examine the specificity of the *PRKACB* (GCC)-repeat inert effect, we performed DNA reconstruction of additional GCC/GGC-repeats in the 5′ UTR of the *SMAD9* and *RASGEF1C* genes, and also a non-GCC STR, such as a GA-repeat in the 5′ UTR of human *GPM6B* (Supplementary 3). Strikingly, while the reconstructed 3D structures were significantly divergent at various lengths in the instance of the *GPM6B* 5′ UTR GA-repeat, the *SMAD9* and *RASGEF1C* 3D structures were almost identical for all repeat lengths studied. We also studied the interactive effect of divergent non-repeat flanking sequences with the (GCC)-repeats by studying a different species than human, such as capuchin. The inert effect of the (GCC)-block was consistent, regardless of the differential flanking non-repeat sequences in capuchin versus human.

### Gene network reconstruction

**Figure 5:**
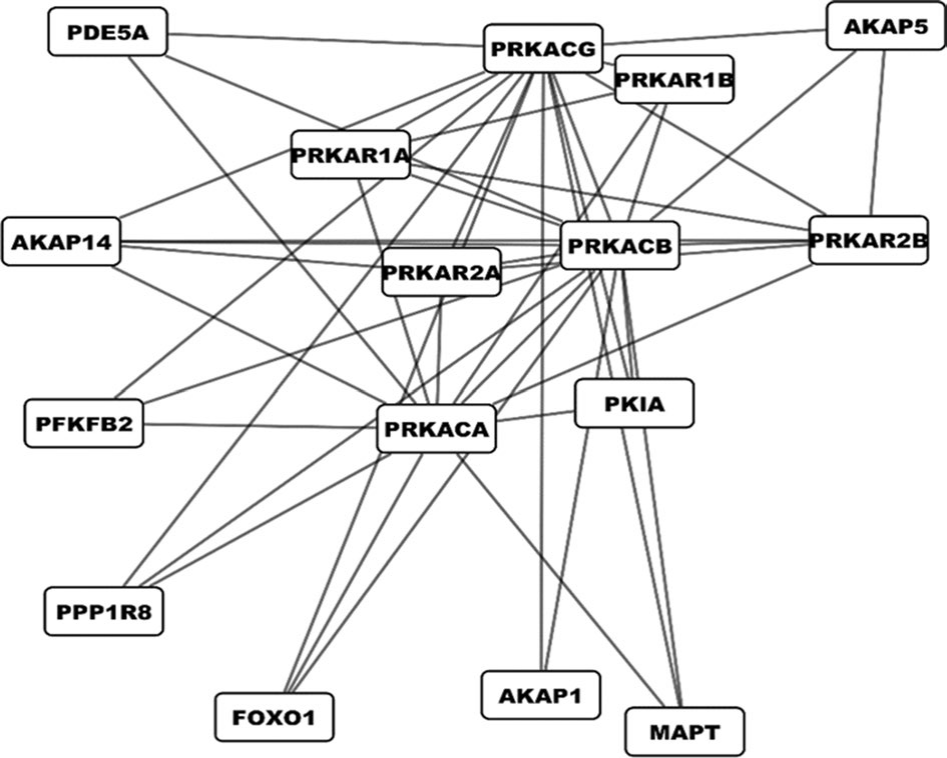
The interactive network reconstructed based on experimental evidence. The network consists of 16 nodes, including genes of the cAMP-dependent protein kinase subunits, as well as other structurally and functionally important genes such as MAPT, FOXO1, and PDE5A. The 16 genes interact with each other through 45 edges.

Based on the available experimental evidence, the reconstructed interactive network consisted of 16 nodes and 45 edges (Fig. 5). Within this network, *PRKACB* interacts with genes of other subunits of the cAMP-dependent protein kinase complex, as well as other genes such as *MAPT*, *FOXO1*, and *PDE5A*, which are central to maintaining cell structure and function.

## Discussion

Because of containing internal CpG dinucleotides, susceptible to methylation and subsequent base substitutions, (GCC)-repeats are potential mutation hotspots^27–29^, and this may be the reason why this class of repeats do not expand beyond certain lengths unless where selected, such as in the gene regulatory regions^30^. Large expansions of (GCC)-repeats in the 5′ UTR of a number of genes are associated with hypermethylation and intellectual disability^31^. Based on the Ensembl database, the *PRKACB* gene is in the 10th percentile of genes in respect of containing long (GCC)-repeats^14^. Here we show that this (GCC)-repeat is predominantly monomorphic in human at 7-repeats, and there may be enrichment of divergence from this monomorphism in late-onset NCD. We propose that there was selective advantage in human at this particular STR to reach to, and stabilize at that length. The above proposition is supported by the observations that mutations that have negative fitness consequences tend to be eliminated from the population^32^. This STR was found to be highly conserved across various mammalian species, indicating its important role in growth and development. In general, STRs near the TSSs of genes are often highly conserved, and distance from a STR to the nearest TSS is a good predictor of the STR conservation score^33^.

We also found that (GCC)7 periodicals had the least effect on the 3D structure in contrast to the more significant effects that various other repeats inserted, which probably confers an inert effect to (GCC)7 without changing the structure of DNA or RNA. The above was deduced from the exceptionally continuous trend of those periodicals (uninterrupted by high divergence scores) in contrast to all other repeats. Interestingly, the divergence score between the 7 and 8 repeat (the latter was an allele of the two divergent genotypes) was among the highest in all examined models. While we also detected an inert effect of GCC/GGC repeats in two additional genes, *SMAD9* and *RASGEF1C*, this effect was not observed in the GA class of STRs, where various lengths of GA-repeats resulted in significant variation in the 3D structure of DNA in the present study and in a previously reported research in the instance of human *ZMYM3*^11^. Significant divergence has also been observed with various CA-repeat lengths in the human *NHLH2* gene^9^. The above findings indicate that long GCC/GGC repeats might have evolved to exert epigenetic effects, as epigenetic knobs, without changing the structure of the regional DNA.

GCC motifs of STRs significantly overlap with G-quadraplex (G4) non-B structures^34^. Recent research indicates that organisms may have evolutionarily developed G4 into a novel reversible and elaborate transcriptional regulatory mechanism benefiting multiple physiological activities of higher organisms^35,36^.

*PRKACB* is involved in a number of physiological and pathological pathways, the outcomes of which directly affect cognition in human^12,13,37–39^. Future studies are warranted to sequence the *PRKACB* (GCC)-repeat in a large number of patients with neurological disorders. Considering the pivotal role of *PRKACB* in the brain, CRISPR/Cas9 methods may also be ideal to edit this STR at the genomic level, and investigate differentiation of human stem cells into neural cells.

## Conclusion

In conclusion, we report the first instance of predominant monomorphism of a long (GCC)-repeat in human and enriched divergent genotypes from this monomorphism in human disease. We also propose that (GCC)-repeats might have evolved in the genome as regulatory elements (such as epigenetic regulation) without dramatically changing the 3D structure of the regional DNA. Further experimental research is warranted to examine the proposed model.

## Supplementary Information

Supplementary Information.

## Abbreviations

3D: Three-dimensional
NCD: Neurocognitive disorder
PRKACB: Protein Kinase CAMP-Activated Catalytic Subunit Beta
STR: Short tandem repeat
TSS: Transcription start site
UTR: Untranslated region
