# Supplementary material for: Novel implications of a strictly monomorphic (GCC) repeat in the human PRKACB gene
Source: Sci Rep. 2021 Oct 19;11:20629. doi: 10.1038/s41598-021-99932-3 (PMC8526596; doi:10.1038/s41598-021-99932-3)
Supplement: Supplementary file 1 — Supplementary Information. [file 41598_2021_99932_MOESM1_ESM.pdf]

Suppl. 1. Confirmatory reverse sequencing of the divergent 7/8 genotypes.

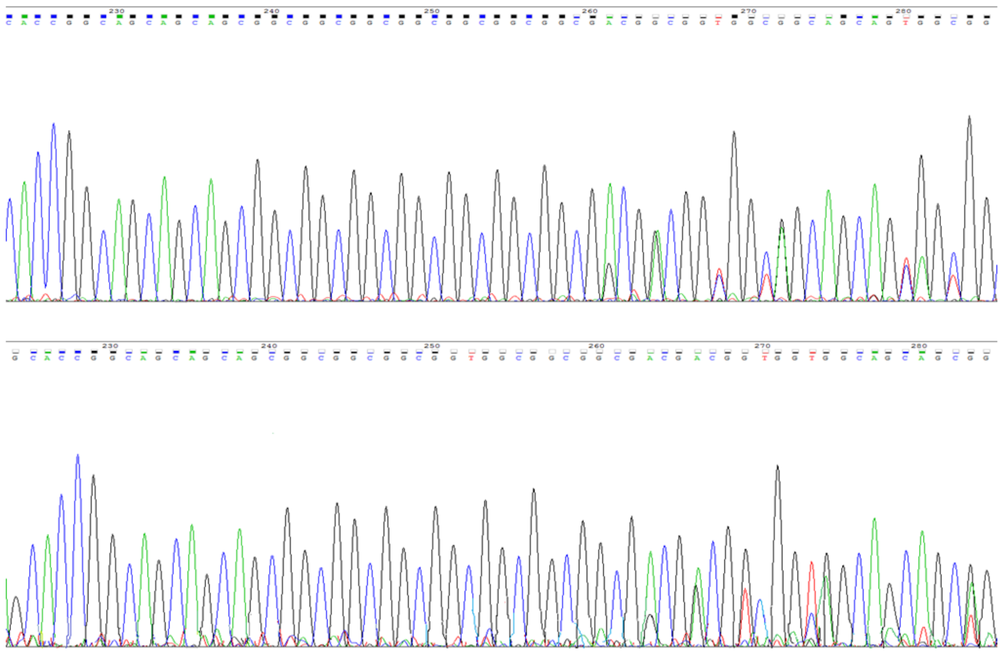

Suppl. 2. Implemented DNA 3D construction models.

AA-Wedge model

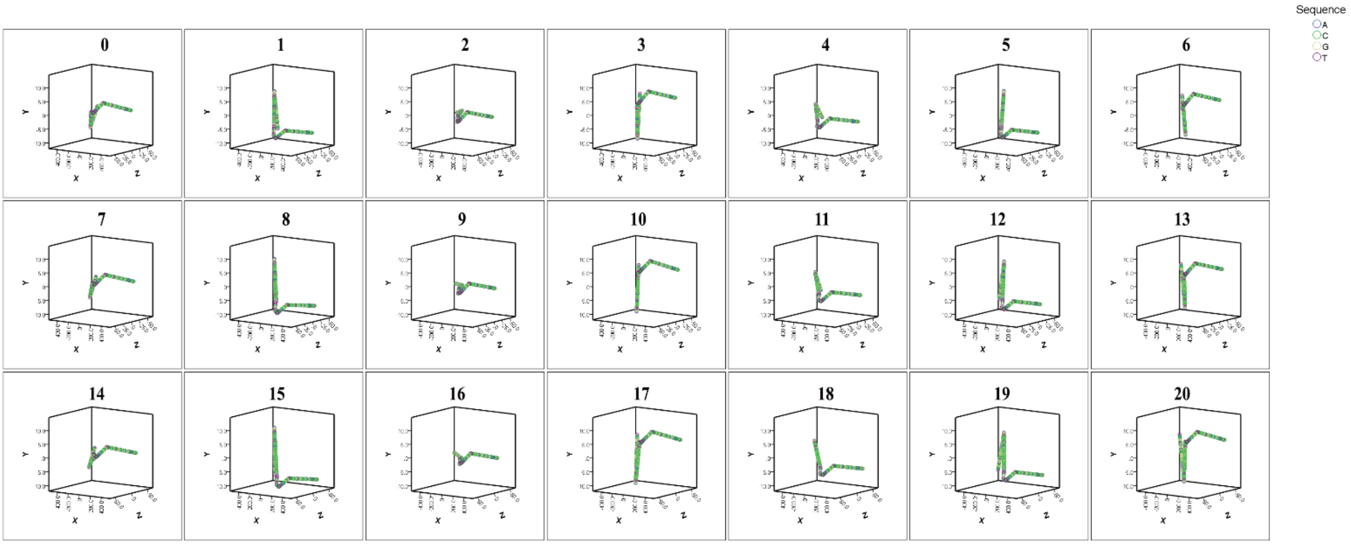

Repeats	0	1	2	3	4	5	6	7	8	9	10	11	12	13	14	15	16	17	18	19	20
0	0.00	0.51	0.37	0.12	0.38	0.57	0.25	0.05	0.52	0.37	0.13	0.43	0.60	0.26	0.09	0.53	0.39	0.14	0.47	0.60	0.27
1	0.51	0.00	0.39	0.53	0.19	0.16	0.57	0.49	0.05	0.44	0.52	0.15	0.20	0.57	0.48	0.09	0.49	0.52	0.14	0.23	0.57
2	0.37	0.39	0.00	0.35	0.31	0.34	0.27	0.35	0.39	0.10	0.34	0.36	0.34	0.27	0.34	0.39	0.20	0.34	0.39	0.35	0.27
3	0.12	0.53	0.35	0.00	0.42	0.56	0.16	0.14	0.53	0.35	0.04	0.47	0.58	0.17	0.17	0.54	0.38	0.07	0.50	0.58	0.19
4	0.38	0.19	0.31	0.42	0.00	0.33	0.48	0.36	0.21	0.37	0.41	0.09	0.36	0.48	0.36	0.24	0.44	0.41	0.14	0.37	0.49
5	0.57	0.16	0.34	0.56	0.33	0.00	0.52	0.56	0.13	0.39	0.55	0.28	0.06	0.52	0.55	0.14	0.46	0.55	0.25	0.09	0.52
6	0.25	0.57	0.27	0.16	0.48	0.52	0.00	0.25	0.57	0.25	0.15	0.53	0.52	0.04	0.27	0.56	0.28	0.17	0.56	0.52	0.06
7	0.05	0.49	0.35	0.14	0.36	0.56	0.25	0.00	0.51	0.35	0.13	0.42	0.58	0.26	0.04	0.52	0.37	0.13	0.45	0.59	0.27
8	0.52	0.05	0.39	0.53	0.21	0.13	0.57	0.51	0.00	0.43	0.53	0.16	0.17	0.57	0.49	0.05	0.49	0.52	0.13	0.19	0.56
9	0.37	0.44	0.10	0.35	0.37	0.39	0.25	0.35	0.43	0.00	0.34	0.39	0.39	0.25	0.32	0.43	0.10	0.33	0.42	0.40	0.25
10	0.13	0.52	0.34	0.04	0.41	0.55	0.15	0.13	0.53	0.34	0.00	0.46	0.57	0.15	0.15	0.53	0.36	0.03	0.49	0.57	0.17
11	0.43	0.15	0.36	0.47	0.09	0.28	0.53	0.42	0.16	0.39	0.46	0.00	0.30	0.53	0.40	0.18	0.44	0.46	0.06	0.31	0.53
12	0.60	0.20	0.34	0.58	0.36	0.06	0.52	0.58	0.17	0.39	0.57	0.30	0.00	0.52	0.57	0.14	0.45	0.56	0.27	0.04	0.51
13	0.26	0.57	0.27	0.17	0.48	0.52	0.04	0.26	0.57	0.25	0.15	0.53	0.52	0.00	0.27	0.56	0.26	0.15	0.55	0.52	0.03
14	0.09	0.48	0.34	0.17	0.36	0.55	0.27	0.04	0.49	0.32	0.15	0.40	0.57	0.27	0.00	0.51	0.34	0.14	0.44	0.57	0.27
15	0.53	0.09	0.39	0.54	0.24	0.14	0.56	0.52	0.05	0.43	0.53	0.18	0.14	0.56	0.51	0.00	0.49	0.52	0.14	0.16	0.56
16	0.39	0.49	0.20	0.38	0.44	0.46	0.28	0.37	0.49	0.10	0.36	0.44	0.45	0.26	0.34	0.49	0.00	0.35	0.46	0.45	0.26
17	0.14	0.52	0.34	0.07	0.41	0.55	0.17	0.13	0.52	0.33	0.03	0.46	0.56	0.15	0.14	0.52	0.35	0.00	0.48	0.57	0.15
18	0.47	0.14	0.39	0.50	0.14	0.25	0.56	0.45	0.13	0.42	0.49	0.06	0.27	0.55	0.44	0.14	0.46	0.48	0.00	0.27	0.55
19	0.60	0.23	0.35	0.58	0.37	0.09	0.52	0.59	0.19	0.40	0.57	0.31	0.04	0.52	0.57	0.16	0.45	0.57	0.27	0.00	0.51
20	0.27	0.57	0.27	0.19	0.49	0.52	0.06	0.27	0.56	0.25	0.17	0.53	0.51	0.03	0.27	0.56	0.26	0.15	0.55	0.51	0.00

DNase model (Lazarovici, Zhou et al. 2013)

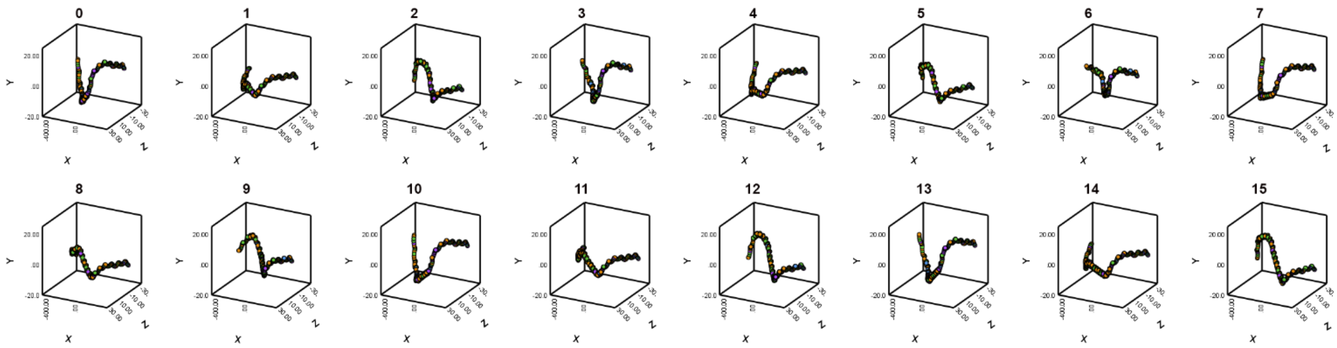

Repeat	0	1	2	3	4	5	6	7	8	9	10	11	12	13	14	15
0	0.00	0.26	0.63	0.13	0.19	0.60	0.26	0.15	0.54	0.53	0.09	0.44	0.63	0.12	0.29	0.62
1	0.26	0.00	0.55	0.31	0.11	0.46	0.38	0.15	0.16	0.53	0.21	0.24	0.59	0.29	0.11	0.53
2	0.63	0.55	0.00	0.58	0.61	0.17	0.51	0.62	0.27	0.16	0.61	0.42	0.13	0.57	0.55	0.10
3	0.13	0.31	0.58	0.00	0.25	0.58	0.25	0.22	0.55	0.43	0.16	0.45	0.56	0.08	0.33	0.58
4	0.19	0.11	0.61	0.25	0.00	0.52	0.35	0.08	0.44	0.55	0.14	0.16	0.64	0.23	0.23	0.59
5	0.60	0.46	0.17	0.58	0.52	0.00	0.54	0.56	0.14	0.39	0.57	0.29	0.16	0.56	0.45	0.14
6	0.26	0.38	0.51	0.25	0.35	0.54	0.00	0.32	0.54	0.32	0.26	0.47	0.48	0.17	0.39	0.52
7	0.15	0.15	0.62	0.22	0.08	0.56	0.32	0.00	0.48	0.55	0.09	0.35	0.65	0.19	0.17	0.61
8	0.54	0.16	0.27	0.55	0.44	0.14	0.54	0.48	0.00	0.43	0.51	0.19	0.35	0.53	0.36	0.16
9	0.53	0.53	0.16	0.43	0.55	0.39	0.32	0.55	0.43	0.00	0.52	0.45	0.29	0.44	0.51	0.32
10	0.09	0.21	0.61	0.16	0.14	0.57	0.26	0.09	0.51	0.52	0.00	0.39	0.62	0.12	0.22	0.60
11	0.44	0.24	0.42	0.45	0.16	0.29	0.47	0.35	0.19	0.45	0.39	0.00	0.48	0.43	0.22	0.39
12	0.63	0.59	0.13	0.56	0.64	0.16	0.48	0.65	0.35	0.29	0.62	0.48	0.00	0.56	0.59	0.17
13	0.12	0.29	0.57	0.08	0.23	0.56	0.17	0.19	0.53	0.44	0.12	0.43	0.56	0.00	0.29	0.57
14	0.29	0.11	0.55	0.33	0.23	0.45	0.39	0.17	0.36	0.51	0.22	0.22	0.59	0.29	0.00	0.52
15	0.62	0.53	0.10	0.58	0.59	0.14	0.52	0.61	0.16	0.32	0.60	0.39	0.17	0.57	0.52	0.00

Bolshoy (Bolshoy, McNamara et al. 1991)

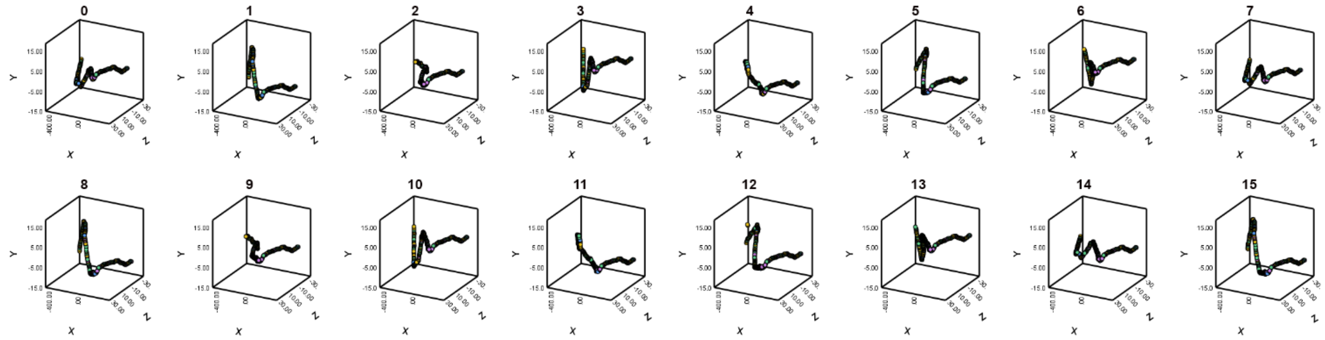

Repeat	0	1	2	3	4	5	6	7	8	9	10	11	12	13	14	15
0	0.00	0.61	0.31	0.16	0.58	0.58	0.23	0.11	0.61	0.29	0.18	0.59	0.56	0.26	0.18	0.60
1	0.61	0.00	0.46	0.64	0.14	0.14	0.64	0.60	0.08	0.47	0.62	0.16	0.18	0.65	0.57	0.14
2	0.31	0.46	0.00	0.27	0.52	0.39	0.21	0.30	0.46	0.09	0.27	0.52	0.39	0.25	0.30	0.46
3	0.16	0.64	0.27	0.00	0.66	0.60	0.10	0.15	0.64	0.26	0.08	0.66	0.58	0.13	0.20	0.63
4	0.58	0.14	0.52	0.66	0.00	0.24	0.69	0.58	0.14	0.53	0.64	0.08	0.25	0.70	0.56	0.17
5	0.58	0.14	0.39	0.60	0.24	0.00	0.58	0.57	0.13	0.40	0.58	0.25	0.08	0.58	0.54	0.15
6	0.23	0.64	0.21	0.10	0.69	0.58	0.00	0.21	0.64	0.20	0.12	0.69	0.57	0.07	0.24	0.63
7	0.11	0.60	0.30	0.15	0.58	0.57	0.21	0.00	0.60	0.27	0.14	0.58	0.55	0.23	0.10	0.59
8	0.61	0.08	0.46	0.64	0.14	0.13	0.64	0.60	0.00	0.47	0.62	0.13	0.13	0.65	0.57	0.07
9	0.29	0.47	0.09	0.26	0.53	0.40	0.20	0.27	0.47	0.00	0.24	0.53	0.39	0.21	0.27	0.47
10	0.18	0.62	0.27	0.08	0.64	0.58	0.12	0.14	0.62	0.24	0.00	0.64	0.57	0.11	0.15	0.62
11	0.59	0.16	0.52	0.66	0.08	0.25	0.69	0.58	0.13	0.53	0.64	0.00	0.24	0.70	0.56	0.13
12	0.56	0.18	0.39	0.58	0.25	0.08	0.57	0.55	0.13	0.39	0.57	0.24	0.00	0.58	0.53	0.13
13	0.26	0.65	0.25	0.13	0.70	0.58	0.07	0.23	0.65	0.21	0.11	0.70	0.58	0.00	0.23	0.64
14	0.18	0.57	0.30	0.20	0.56	0.54	0.24	0.10	0.57	0.27	0.15	0.56	0.53	0.23	0.00	0.57
15	0.60	0.14	0.46	0.63	0.17	0.15	0.63	0.59	0.07	0.47	0.62	0.13	0.13	0.64	0.57	0.00

Cacchione model (Cacchione, De Santis et al. 1989)

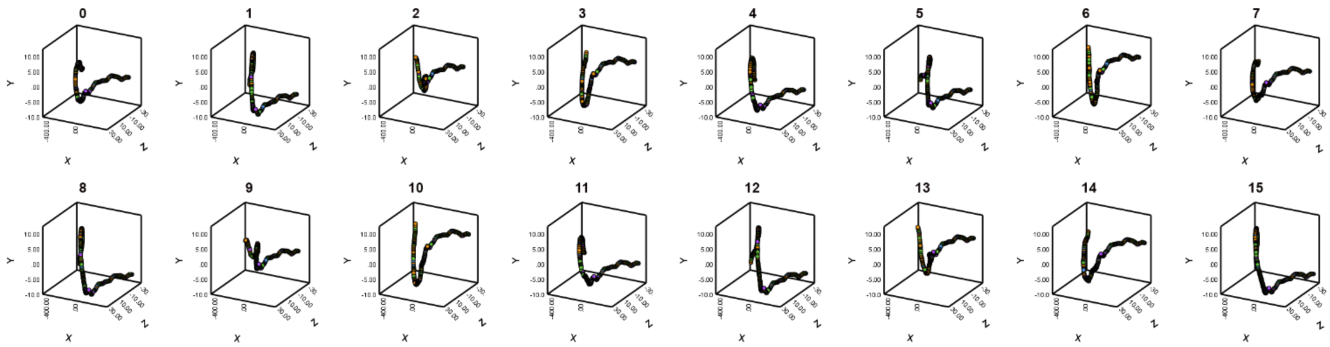

Repeat	0	1	2	3	4	5	6	7	8	9	10	11	12	13	14	15
0	0.00	0.41	0.48	0.33	0.27	0.48	0.44	0.21	0.37	0.48	0.39	0.18	0.45	0.48	0.31	0.32
1	0.41	0.00	0.59	0.60	0.17	0.12	0.61	0.54	0.08	0.50	0.60	0.27	0.09	0.59	0.57	0.14
2	0.48	0.59	0.00	0.24	0.63	0.54	0.09	0.33	0.62	0.19	0.17	0.59	0.58	0.06	0.26	0.63
3	0.33	0.60	0.24	0.00	0.54	0.62	0.17	0.13	0.59	0.37	0.10	0.45	0.62	0.26	0.07	0.55
4	0.27	0.17	0.63	0.54	0.00	0.24	0.61	0.44	0.12	0.58	0.57	0.13	0.21	0.62	0.51	0.09
5	0.48	0.12	0.54	0.62	0.24	0.00	0.58	0.58	0.17	0.41	0.60	0.33	0.10	0.53	0.59	0.22
6	0.44	0.61	0.09	0.17	0.61	0.58	0.00	0.27	0.62	0.26	0.09	0.56	0.60	0.10	0.19	0.61
7	0.21	0.54	0.33	0.13	0.44	0.58	0.27	0.00	0.51	0.40	0.20	0.34	0.56	0.33	0.12	0.46
8	0.37	0.08	0.62	0.59	0.12	0.17	0.62	0.51	0.00	0.55	0.60	0.22	0.11	0.61	0.55	0.07
9	0.48	0.50	0.19	0.37	0.58	0.41	0.26	0.40	0.55	0.00	0.31	0.57	0.48	0.18	0.34	0.58
10	0.39	0.60	0.17	0.10	0.57	0.60	0.09	0.20	0.60	0.31	0.00	0.50	0.60	0.17	0.10	0.58
11	0.18	0.27	0.59	0.45	0.13	0.33	0.56	0.34	0.22	0.57	0.50	0.00	0.30	0.58	0.43	0.17
12	0.45	0.09	0.58	0.62	0.21	0.10	0.60	0.56	0.11	0.48	0.60	0.30	0.00	0.57	0.58	0.15
13	0.48	0.59	0.06	0.26	0.62	0.53	0.10	0.33	0.61	0.18	0.17	0.58	0.57	0.00	0.24	0.61
14	0.31	0.57	0.26	0.07	0.51	0.59	0.19	0.12	0.55	0.34	0.10	0.43	0.58	0.24	0.00	0.52
15	0.32	0.14	0.63	0.55	0.09	0.22	0.61	0.46	0.07	0.58	0.58	0.17	0.15	0.61	0.52	0.00

Calladine model (Calladine, Drew et al. 1988)

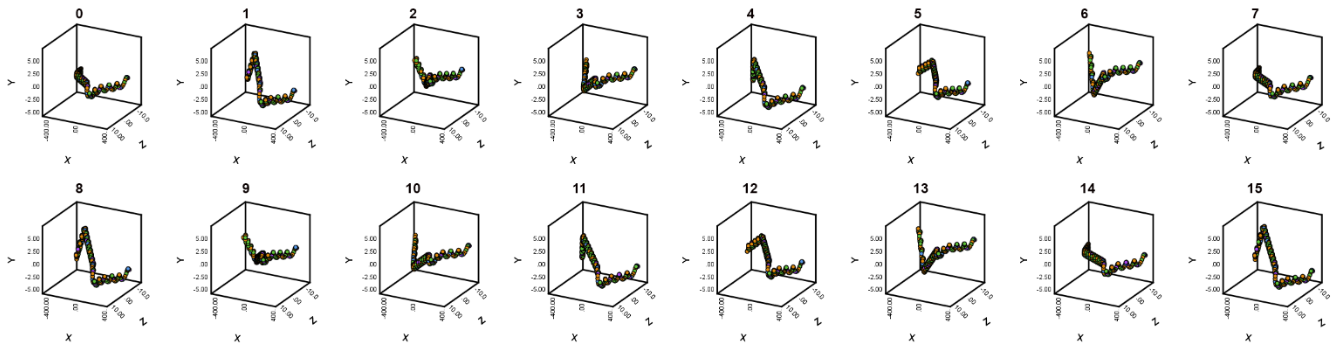

Repeat	0	1	2	3	4	5	6	7	8	9	10	11	12	13	14	15
0	0.00	0.25	0.51	0.40	0.18	0.32	0.47	0.17	0.27	0.51	0.41	0.19	0.33	0.48	0.21	0.28
1	0.25	0.00	0.50	0.51	0.15	0.19	0.52	0.28	0.08	0.50	0.52	0.16	0.20	0.52	0.32	0.10
2	0.51	0.50	0.00	0.23	0.55	0.42	0.16	0.49	0.49	0.11	0.24	0.54	0.42	0.16	0.47	0.49
3	0.40	0.51	0.23	0.00	0.47	0.49	0.17	0.38	0.51	0.28	0.12	0.46	0.49	0.19	0.37	0.51
4	0.18	0.15	0.55	0.47	0.00	0.28	0.52	0.22	0.16	0.56	0.48	0.08	0.29	0.53	0.25	0.18
5	0.32	0.19	0.42	0.49	0.28	0.00	0.47	0.31	0.19	0.42	0.50	0.28	0.13	0.46	0.34	0.18
6	0.47	0.52	0.16	0.17	0.52	0.47	0.00	0.44	0.51	0.17	0.14	0.51	0.48	0.08	0.43	0.51
7	0.17	0.28	0.49	0.38	0.22	0.31	0.44	0.00	0.27	0.50	0.40	0.20	0.32	0.46	0.18	0.29
8	0.27	0.08	0.49	0.51	0.16	0.19	0.51	0.27	0.00	0.49	0.52	0.15	0.17	0.51	0.31	0.08
9	0.51	0.50	0.11	0.28	0.56	0.42	0.17	0.50	0.49	0.00	0.23	0.55	0.42	0.16	0.48	0.49
10	0.41	0.52	0.24	0.12	0.48	0.50	0.14	0.40	0.52	0.23	0.00	0.47	0.49	0.16	0.38	0.52
11	0.19	0.16	0.54	0.46	0.08	0.28	0.51	0.20	0.15	0.55	0.47	0.00	0.26	0.52	0.24	0.16
12	0.33	0.20	0.42	0.49	0.29	0.13	0.48	0.32	0.17	0.42	0.49	0.26	0.00	0.47	0.33	0.17
13	0.48	0.52	0.16	0.19	0.53	0.46	0.08	0.46	0.51	0.16	0.16	0.52	0.47	0.00	0.44	0.51
14	0.21	0.32	0.47	0.37	0.25	0.34	0.43	0.18	0.31	0.48	0.38	0.24	0.33	0.44	0.00	0.31
15	0.28	0.10	0.49	0.51	0.18	0.18	0.51	0.29	0.08	0.49	0.52	0.16	0.17	0.51	0.31	0.00

References

Bolshoy, A., P. McNamara, R. Harrington and E. Trifonov (1991). "Curved DNA without A-A: Experimental estimation of all 16 DNA wedge angles." Proceedings of the National Academy of Sciences of the United States of America **88**: 2312-2316.

Cacchione, S., P. De Santis, D. Foti, A. Palleschi and M. Savino (1989). "Periodical polydeoxynucleotides and DNA curvature." Biochemistry **28**(22): 8706-8713.

Calladine, C. R., H. R. Drew and M. J. McCall (1988). "The intrinsic curvature of DNA in solution." Journal of Molecular Biology **201**(1): 127-137.

Fratini, A. V., M. L. Kopka, H. R. Drew and R. E. Dickerson (1982). "Reversible bending and helix geometry in a B-DNA dodecamer: CGCGAATTBrCGCG." J Biol Chem **257**(24): 14686-14707.

Lazarovici, A., T. Zhou, A. Shafer, A. C. Dantas Machado, T. R. Riley, R. Sandstrom, P. J. Sabo, Y. Lu, R. Rohs, J. A. Stamatoyannopoulos and H. J. Bussemaker (2013). "Probing DNA shape and methylation state on a genomic scale with DNase I." Proceedings of the National Academy of Sciences **110**(16): 6376-6381.

Suppl. 3. Sample genes containing GCC/GGC repeats and non-GCC repeats for comparison.

Human SMAD9

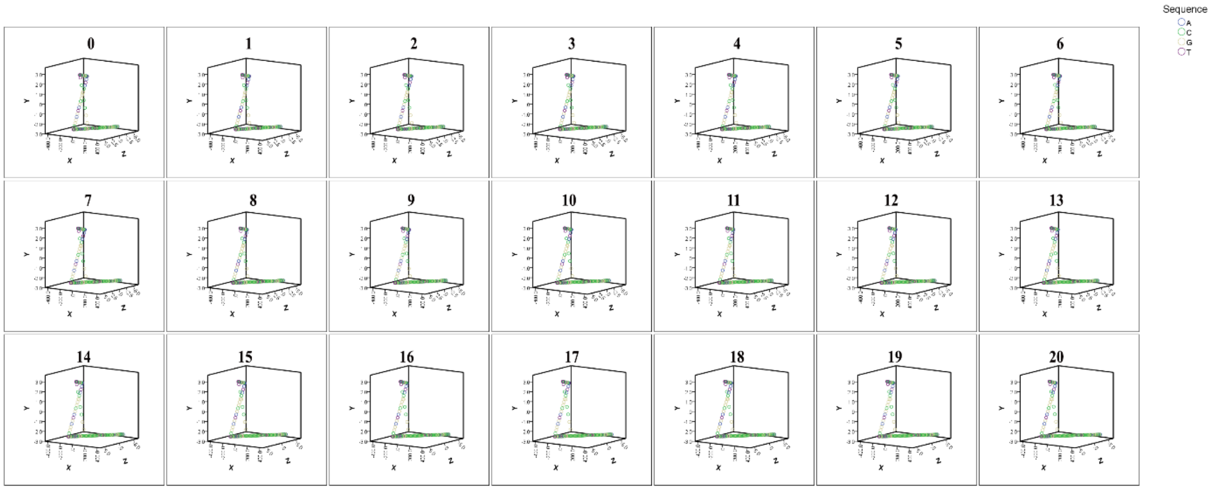

The GCC repeat in the 5' UTR of the above gene resulted in the least divergence in the 3D structure of DNA for all repeats.

Human RASGEF1C

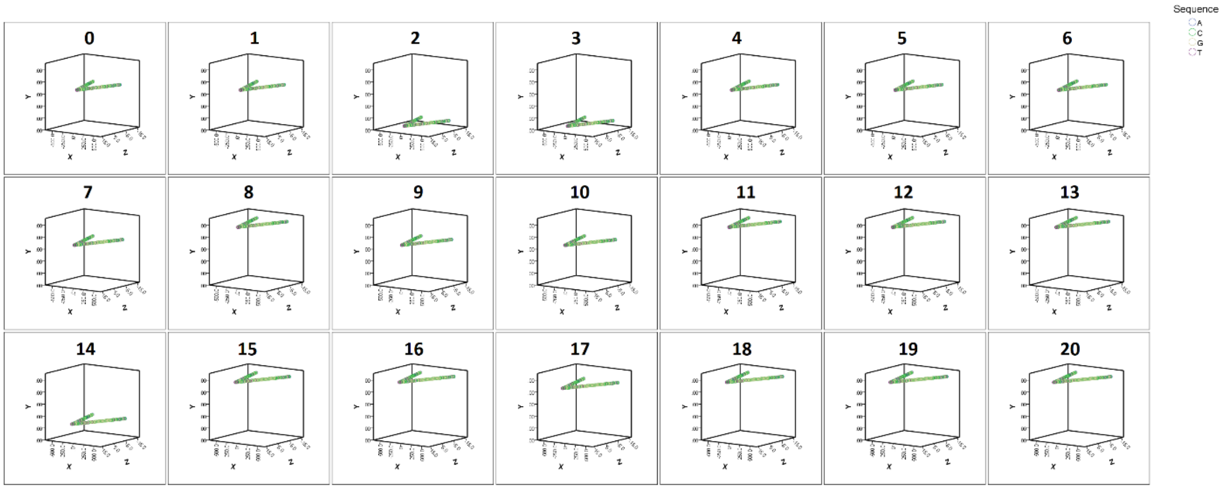

The GGC repeat in the 5' UTR of the above gene resulted in the least divergence in the 3D structure of DNA for all repeats.

Human GPM6B

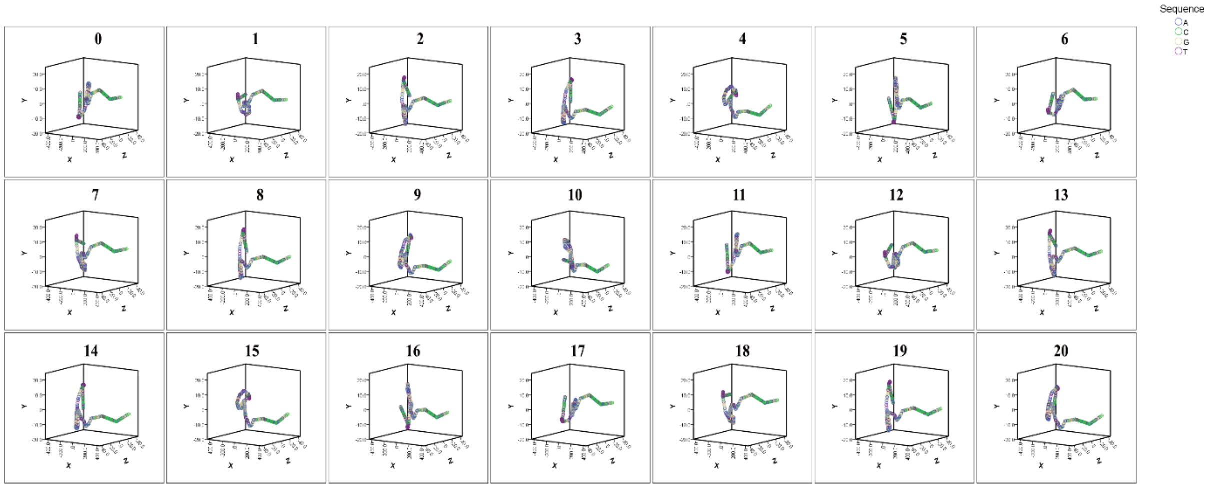

GA-repeats of all lengths exert significant effects on the 3D structure of the above gene.

Capuchin PRKACB

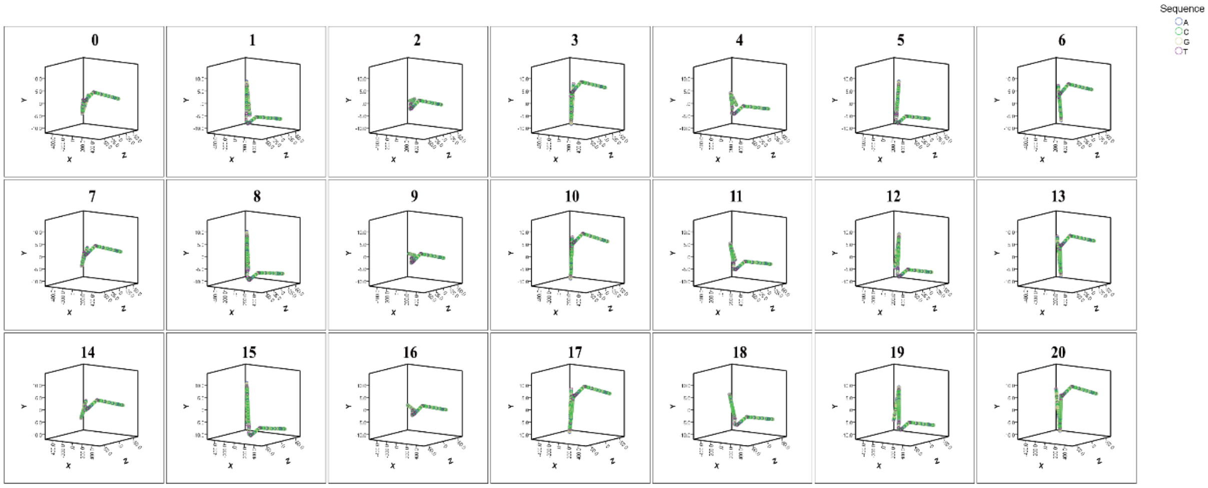

The GCC repeat in the 5’ UTR of a different species from human, which contained diverged flanking sequences resulted in similar results to human (least divergence effect on the 3D structure with 7-repeat additional or subtraction), indicating that the effect of GCC repeats is dominant to the effect of the flanking non-repeat sequences.
